# Developmental plasticity of spatial hearing following asymmetric hearing loss: context-dependent cue integration and its clinical implications

**DOI:** 10.3389/fnsys.2013.00123

**Published:** 2013-12-27

**Authors:** Peter Keating, Andrew J. King

**Affiliations:** Department of Physiology, Anatomy and Genetics, University of OxfordOxford, UK

**Keywords:** auditory localization, binaural, monaural, conductive hearing loss, adaptation, learning, cortex, midbrain

## Abstract

Under normal hearing conditions, comparisons of the sounds reaching each ear are critical for accurate sound localization. Asymmetric hearing loss should therefore degrade spatial hearing and has become an important experimental tool for probing the plasticity of the auditory system, both during development and adulthood. In clinical populations, hearing loss affecting one ear more than the other is commonly associated with otitis media with effusion, a disorder experienced by approximately 80% of children before the age of two. Asymmetric hearing may also arise in other clinical situations, such as after unilateral cochlear implantation. Here, we consider the role played by spatial cue integration in sound localization under normal acoustical conditions. We then review evidence for adaptive changes in spatial hearing following a developmental hearing loss in one ear, and show that adaptation may be achieved either by learning a new relationship between the altered cues and directions in space or by changing the way different cues are integrated in the brain. We next consider developmental plasticity as a source of vulnerability, describing maladaptive effects of asymmetric hearing loss that persist even when normal hearing is provided. We also examine the extent to which the consequences of asymmetric hearing loss depend upon its timing and duration. Although much of the experimental literature has focused on the effects of a stable unilateral hearing loss, some of the most common hearing impairments experienced by children tend to fluctuate over time. We therefore propose that there is a need to bridge this gap by investigating the effects of recurring hearing loss during development, and outline recent steps in this direction. We conclude by arguing that this work points toward a more nuanced view of developmental plasticity, in which plasticity may be selectively expressed in response to specific sensory contexts, and consider the clinical implications of this.

## Introduction

The ability to hear is of critical importance for a wide variety of species. Indeed, in many naturalistic situations, auditory input provides the only source of information about distant events. However, whilst the identity of a sound source is clearly important, its location also plays a critical role in guiding behavior. In noisy and reverberant acoustic environments, spatial hearing can additionally help to separate different sound sources, thereby enabling their subsequent identification (Yost, 1997; Kidd et al., 2005). For these reasons, numerous species have developed and refined the ability to localize sounds in space.

Unlike the visual and somatosensory systems, however, the auditory system does not contain an implicit map of space at the level of the receptor surface. Instead, the receptors that transduce sound are arranged along the cochlea according to their tuning for sound frequency. The brain must therefore actively construct a representation of auditory space by transforming and processing the acoustical inputs provided to each ear. In doing so, the brain takes advantage of the fact that specific aspects of the acoustical input tend to depend on the position of the sound source relative to that of the listener (Blauert, 1997). The challenge faced by the brain is therefore to interpret and combine the information provided by these different spatial cues in order to create a coherent representation of auditory space.

In many cases, the most effective way to localize sounds will depend on the precise properties of the acoustical environment. While this varies with the nature of the sound sources themselves and the acoustical conditions in which they are encountered, developmental changes in the relative dimensions of the ears and in the neural circuits that process sound will also cause the acoustical environment to change. In order to maintain stable representations of space, the auditory system must therefore adapt to these changes by processing auditory spatial cues dynamically in ways that are appropriate to the prevailing sensory conditions. To understand these adaptive mechanisms, one popular approach that has been used is to manipulate the acoustical input experimentally and study its consequences on the perception and processing of sound. Although the sounds reaching the ears can be altered in a variety of ways, important insights into developmental plasticity have been gained by introducing a hearing loss to one ear (Clopton and Silverman, 1977; Silverman and Clopton, 1977; Clements and Kelly, 1978; Moore and Irvine, 1981; Brugge et al., 1985; Popescu and Polley, 2010; Keating et al., 2013a; Polley et al., 2013). In this way, asymmetric hearing loss has become an important model system for understanding basic principles of neural development, complementing studies of monocular deprivation in the visual system (Daw, 2009).

However, whilst monaural occlusion provides a powerful method for studying basic aspects of developmental plasticity, the developmental effects of asymmetric hearing loss are also clinically important. This is because periods of unilateral hearing loss are extremely common during development. For example, otitis media with effusion, colloquially referred to as “glue ear,” is experienced by approximately 80% of children before the age of 3, and is often associated with a temporary hearing loss in one ear (Engel et al., 1999; Whitton and Polley, 2011). In rarer cases, children may also experience a congenital hearing loss in one ear (Wilmington et al., 1994; Gray et al., 2009). Similarly, in situations where children with bilateral deafness receive a cochlear implant in only one ear, the auditory system may be exposed to long periods of unilateral stimulation. This can result in marked changes in auditory pathway circuitry, with important implications for the restoration of normal functions if the second ear is subsequently implanted (Gordon et al., 2013; Illg et al., 2013; Kral et al., 2013). From both a fundamental and clinical perspective, it is therefore extremely important to understand the developmental consequences of asymmetric hearing loss.

In this review, we briefly outline auditory spatial processing under normal acoustical conditions, highlighting the importance of integrating the information provided by the different cues to sound source location. We then review evidence for adaptive changes in spatial hearing following a developmental hearing loss in one ear, and argue that adaptation may be achieved either by learning to use altered cues correctly or by learning to change the way cues are integrated. Having outlined the positive aspects of developmental plasticity, we next consider this plasticity as a source of vulnerability, describing evidence for effects of asymmetric hearing loss that persist and become maladaptive when normal hearing is restored. We then ask whether the consequences of asymmetric hearing loss are mediated by its timing and duration, and suggest that spatial hearing may be particularly vulnerable to prolonged periods of imbalanced hearing early in development.

Although much of the experimental literature has focused on the effects of a stable hearing loss in one ear, some of the most common forms of hearing loss experienced by children tend to fluctuate over time (Hogan et al., 1997; Whitton and Polley, 2011). We therefore propose that there is a need to bridge this gap by investigating the effects of recurring hearing loss during development, and outline recent steps in this direction. In addition to its clinical relevance, we suggest that this approach may also provide a useful experimental tool for understanding how the brain learns the importance of sensory context in complex environments. We then conclude by arguing that this work points toward a more nuanced view of developmental plasticity following asymmetric hearing loss, in which plasticity may be selectively expressed in response to specific hearing conditions.

## Auditory spatial processing under normal acoustical conditions

### Auditory spatial cues

**Figure 1:**
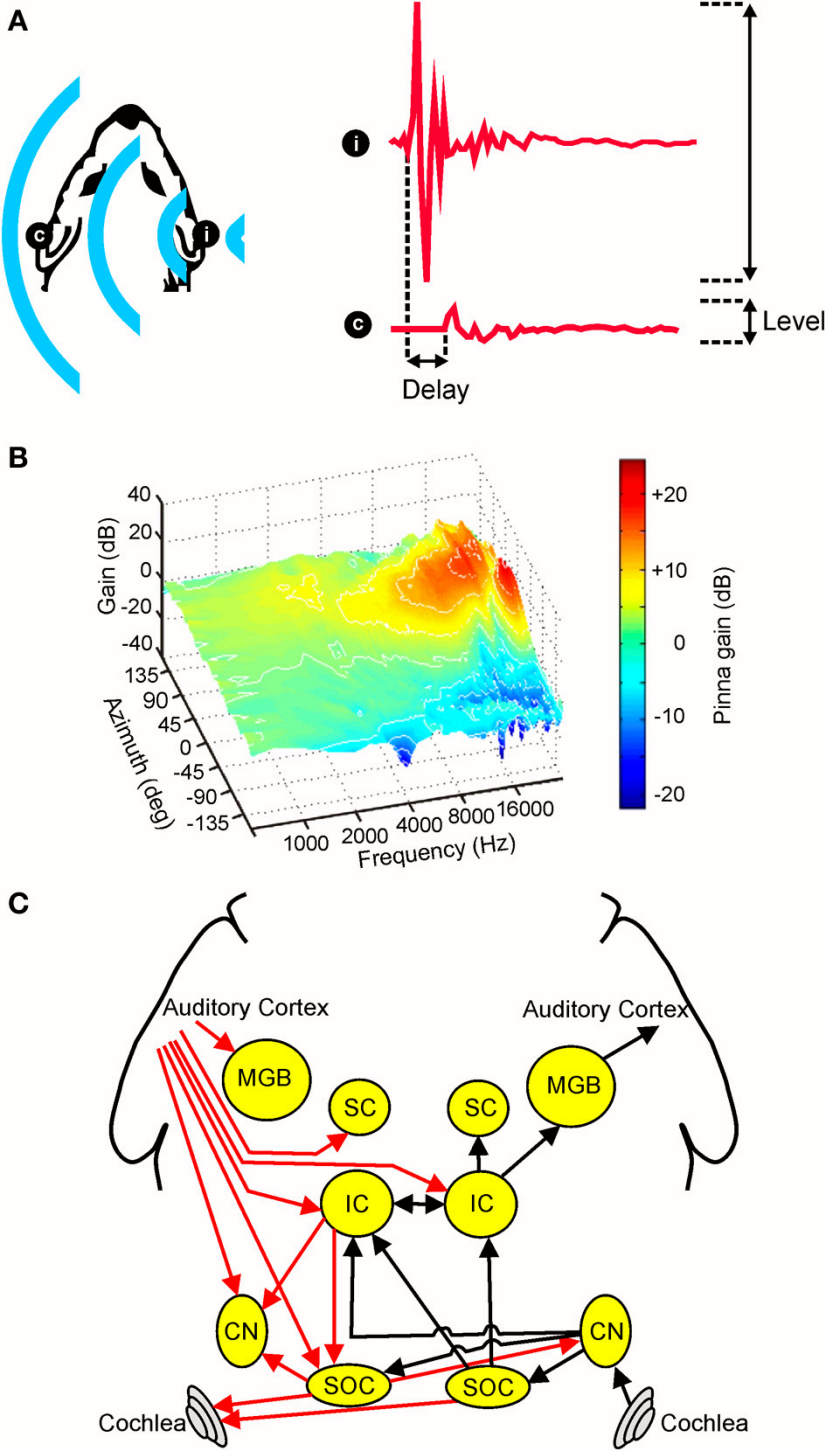
**Overview of auditory spatial processing. (A)** Sample acoustic waveforms are shown for the ipsilateral (i) and contralateral (c) ears following presentation of sound from a source located to one side of the head. The incoming sound is typically delayed and attenuated in the contralateral ear, producing interaural time differences (ITDs) and interaural level differences (ILDs), respectively. These cues are referred to as binaural spatial cues as they depend on comparisons between the two ears. **(B)** Gain is plotted for an adult ferret ear as a function of sound frequency and azimuth. Because the filtering effects of the head and ears depend on the direction of a sound source, the observed spectra vary with respect to azimuth (and elevation), producing spectral shape cues to sound location. **(C)** Simplified schematic of the mammalian auditory pathway showing the principal ascending (black) and descending (red) projections between the cochlea, cochlear nuclei (CN), superior olivary complex (SOC), inferior colliculus (IC), superior colliculus (SC), medial geniculate body (MGB), and auditory cortex. For clarity, each of these projections is shown for one side of the brain only. **(B)** adapted with permission from King et al. (2001).

In individuals with normal hearing, the location of a sound can be inferred from a variety of different cues. However, whilst a complete description of sound source location requires information about both distance and direction, relatively little is known about the neural mechanisms underlying distance perception (Zahorik et al., 2005; Kopčo et al., 2012). For this reason, this review will focus on how the direction of a sound source is computed. In this respect, it has long been known that sounds originating on one side of space tend to be more attenuated in the ear contralateral to the source (Figure 1A). This acoustical shadowing effect of the head produces an interaural level difference (ILD) that varies systematically with sound source direction (Blauert, 1997). Similarly, due to differences in path length between a sound source and the two ears, the acoustic waveform of a sound will often arrive at one ear slightly before the other (Figure 1A). This produces an interaural time difference (ITD) that also varies systematically with the angular direction of the source relative to the head (Blauert, 1997).

Because ITDs and ILDs both require a comparison of the input provided to the two ears, these cues are collectively referred to as binaural spatial cues. In many instances, however, the relative usefulness of these binaural cues depends on sound frequency (Strutt, 1907; Middlebrooks and Green, 1991; Blauert, 1997; Macpherson and Middlebrooks, 2002). For example, since low-frequency sound waves can diffract around the head, ILD cues tend to be relatively small at low frequencies, which limits the usefulness of these cues. This means that ILDs tend to be used primarily for localizing high-frequency sounds.

Conversely, for simple periodic sounds, ITDs become spatially ambiguous as the sound frequency is increased, as it becomes harder to tell at which ear the sound is leading and at which it is lagging (Schnupp et al., 2011). Moreover, ITDs can only be calculated in situations where information is preserved in the auditory system about the temporal structure of the auditory waveform, a feat that many species find difficult to achieve at higher frequencies. This is because auditory nerve fibers represent temporal structure by locking their activity to specific phases of the stimulus waveform. In many species, phase locking begins to decline at frequencies greater than 1 kHz (Sumner and Palmer, 2012), which produces a corresponding reduction in ITD sensitivity at these higher frequencies (Brughera et al., 2013). In such cases, any residual sensitivity to ITDs therefore depends on the envelope of a sound (Henning, 1974; Bernstein and Trahiotis, 2002), rather than its fine temporal structure. The frequency dependence of ILD and ITD sensitivity provides the basis for the duplex theory of sound localization (Strutt, 1907), which applies to humans as well as to at least some other mammalian species (Wakeford and Robinson, 1974; Brown et al., 1978; Houben and Gourevitch, 1979; Keating et al., 2013b).

In addition to these binaural cues, spatial information may also be inferred from the relative intensities of different frequency components present at one ear. This is because, at least in mammals, the filtering properties of the head and external ears serve to shape the spectrum of a sound in a direction-dependent way (Figure 1B). Commonly referred to as spectral cues, these monaural spatial cues are most pronounced at high frequencies, and are thought to be critical for distinguishing between locations that produce identical ITD and ILD values (Musicant and Butler, 1984; Musicant et al., 1990; Carlile et al., 2005). For this reason, spectral cues are thought to play an important role in determining whether sounds are located in the front or rear hemifields. In many mammalian species, including humans, spectral cues are also critical for determining the elevation of a sound (Parsons et al., 1999; Carlile et al., 2005; Tollin et al., 2013). Although the acoustical properties of spectral cues make them equally suitable for determining the lateral angle of sounds in the horizontal plane, these cues typically contribute very little to this process, with ITDs and ILDs instead dominating the perceived azimuth under normal listening conditions (Macpherson and Middlebrooks, 2002).

### Importance of cue integration

The findings outlined in the previous section illustrate that the importance of different spatial cues can vary depending on the properties of the sound and the region of space in which it needs to be localized. Consequently, under a particular set of hearing conditions, different cues tend not to contribute equally to judgments of sound location. Determining the weight that should be given to each cue therefore represents a key aspect of sensory processing. Individually, monaural and binaural spatial cues typically provide only partial, and even potentially contradictory, information about stimulus location. This can occur because neural representations are often noisy and imprecise and the nature of these coding errors may be independent for different cues, thereby giving rise to cue conflict. In addition, whereas monaural spectral cues are influenced by the spectrum of a sound (Wightman and Kistler, 1997), binaural cues are much more robust with respect to the source spectrum. In certain situations, the auditory system may therefore misattribute the spectral properties of a sound to the filtering effects of the head and ears (Hofman and Van Opstal, 2002; Keating et al., 2013a), with the result that monaural and binaural cues may indicate that the sound originated from different directions. Over time, the reliability of each cue can also change. The challenge faced by the brain is therefore to determine the best way of combining these different cues to provide a coherent representation of the external world.

This need for cue integration, however, is not unique to the auditory system. Different sensory systems, for example, often provide complementary information about the location of a particular target object or event. By taking into account the information provided by each system, it is therefore possible to achieve a better estimate of object location than would be possible using either system in isolation (Knill and Pouget, 2004; Alais et al., 2010).

Previous studies have shown that this kind of cue integration may be described by a process that takes the weighted average of individual cues, with the weights given to each being proportional to the relative reliability of that cue. It can also be shown that this simple process is statistically optimal under certain conditions (Ernst and Banks, 2002; Alais and Burr, 2004). Although there has been much recent emphasis on multisensory cue integration, models of cue integration have also been applied to the combination of depth cues within the visual system (Jacobs, 1999), as well as the combination of speech cues within the auditory system (Clayards et al., 2008). It is therefore likely that similar models may apply to the integration of auditory spatial cues (Van Wanrooij and Van Opstal, 2007; Keating et al., 2013a).

### Neural basis of spatial hearing

Although the auditory system must ultimately combine the information provided by different spatial cues, monaural and binaural cues are initially processed separately prior to integration at higher levels of the neuroaxis. In mammals, for example, acoustical inputs are transduced into neural signals by cochlear hair cells before being passed via the auditory nerve to the cochlear nuclei, and it is within the dorsal cochlear nuclei that processing of monaural spectral cues is subsequently thought to occur (Young et al., 1992). Projections originating in the ventral divisions of the cochlear nuclei target the superior olive bilaterally (Figure 1C), allowing input from the two ears to converge for the first time. The nature of this convergence is such, however, that the processing of different binaural cues remains segregated to a large extent, with the lateral superior olive (LSO) involved primarily in ILD processing and the medial superior olive (MSO) more associated with the processing of ITDs (Yin, 2002).

Projections from these brainstem nuclei then ascend to the midbrain, where they are thought to target partially overlapping populations of neurons in the central nucleus of the inferior colliculus (ICc) (Loftus et al., 2010). From here, auditory signals are transmitted via the medial geniculate nucleus of the thalamus to the auditory cortex (Figure 1C), which has been shown by inactivation studies to play a critical role in sound localization (Heffner and Heffner, 1990; Malhotra et al., 2004; Nodal et al., 2012). Although cortical cells presumably integrate the information provided by different auditory spatial cues, there is currently very little evidence to suggest that a topographic map of auditory space is constructed at the level of the cortex (Recanzone and Sutter, 2008; Razak, 2011) or indeed at any subcortical level of the primary auditory pathway in mammals [reviewed in Grothe et al., 2010]. A crude map of auditory space has, however, been described in the nucleus of the brachium of the inferior colliculus (nBIC) (Schnupp and King, 1997), which receives a major source of input from the ICc. The nBIC projects topographically to the superior colliculus (SC), where a more refined map of auditory space is found that shows the same topographic order observed for the representation of other sensory modalities (Palmer and King, 1982; King and Hutchings, 1987). Whilst the precise neural architecture invariably differs across species, broadly similar organizational principles apply to the avian brain, with the optic tectum and the external nucleus of the inferior colliculus (ICx) both showing maps of auditory space in the absence of any topographical representation in the forebrain (Cohen and Knudsen, 1999). However, in contrast to mammals, topographic representations of ITDs and ILDs have been found in the brainstem nuclei where these cues are first computed (Singheiser et al., 2012).

## Developmental plasticity of spatial hearing following asymmetric hearing loss

### Types of hearing loss

In attempting to understand developmental plasticity in the auditory system, numerous studies have investigated the effects of early hearing loss. Although hearing loss can be produced in a number of different ways, these can be broadly categorized as being either sensorineural or conductive in nature, each of which has distinct advantages from an experimental perspective. For example, experimental induction of sensorineural hearing loss, which can be achieved via cochlear ablation (Moore and Kowalchuk, 1988), tends to completely abolish both the transduction of sound as well as any spontaneous activity at the site of the lesion (Tucci et al., 1987). Although this form of hearing loss can completely eliminate binaural spatial cues, it is typically irreversible, making it very difficult to determine what happens to the behavioral and neurophysiological representation of the affected ear unless cochlear implants are used. It is also less appropriate as a model for the types of hearing loss that are typically experienced by children during development (Moore and King, 2004; Tollin, 2010; Whitton and Polley, 2011).

In contrast, conductive hearing loss is typically incomplete, producing only a partial attenuation of acoustic input to the affected ear (Moore et al., 1989; Gravel and Wallace, 2000; Kumpik et al., 2010; Lupo et al., 2010). Conductive hearing loss is also often fully reversible and represents an excellent model for the types of hearing loss that are most commonly experienced during development. This is particularly true of otitis media with effusion (Gravel and Wallace, 2000; Whitton and Polley, 2011), which is associated with an accumulation of fluid in the middle ear. In many cases, this prevents the normal transmission of sound by the middle ear, thereby producing a conductive hearing loss.

Although otitis media with effusion can occur either unilaterally or bilaterally (Hogan et al., 1997; Engel et al., 1999), a situation common to many other forms of hearing loss, experimental studies have shown that the effects of a unilateral hearing loss are typically more dramatic than those observed following a bilateral hearing loss (Silverman and Clopton, 1977; Clements and Kelly, 1978; Moore, 2002; Moore and King, 2004; Keuroghlian and Knudsen, 2007; Tollin, 2010; Whitton and Polley, 2011). In addition to its prominence as an experimental model, unilateral hearing loss therefore represents a major source of vulnerability in clinical populations.

From an experimental perspective, a partial hearing loss in one ear can be reversibly induced either by surgical ligation of the ear canal (Silverman and Clopton, 1977; Moore and Irvine, 1981; Brugge et al., 1985; Popescu and Polley, 2010) or by occluding the ear canal with a material that attenuates sound (Knudsen, 1985; Gold and Knudsen, 1999; Kacelnik et al., 2006; Kumpik et al., 2010; Polley et al., 2013). In addition to reducing the input amplitude, these manipulations tend to delay the transmission of sound to the affected ear. Monaural deprivation therefore has a profound effect on both the ITDs and ILDs available to the listener (Moore et al., 1989; Hartley and Moore, 2003; Kumpik et al., 2010; Lupo et al., 2010), which is likely to be shared by the effects of otitis media with effusion in children (Gravel and Wallace, 2000).

Unilateral hearing loss therefore alters the usefulness of auditory spatial cues as well as the precise relationship between specific cue values and spatial location, but leaves any monaural spatial cues available to the intact ear unchanged. The methods used to induce hearing loss also tend to act as low-pass filters, attenuating higher frequencies to a greater extent than lower frequencies (Moore et al., 1989; Kumpik et al., 2010; Lupo et al., 2010; Polley et al., 2013). Consequently, ILDs will typically be altered in a frequency-dependent manner. Although this frequency-dependent attenuation can have profound effects on tonotopic representations in the brain (Popescu and Polley, 2010), this review will focus on its impact on spatial hearing.

### Cue remapping

**Figure 2:**
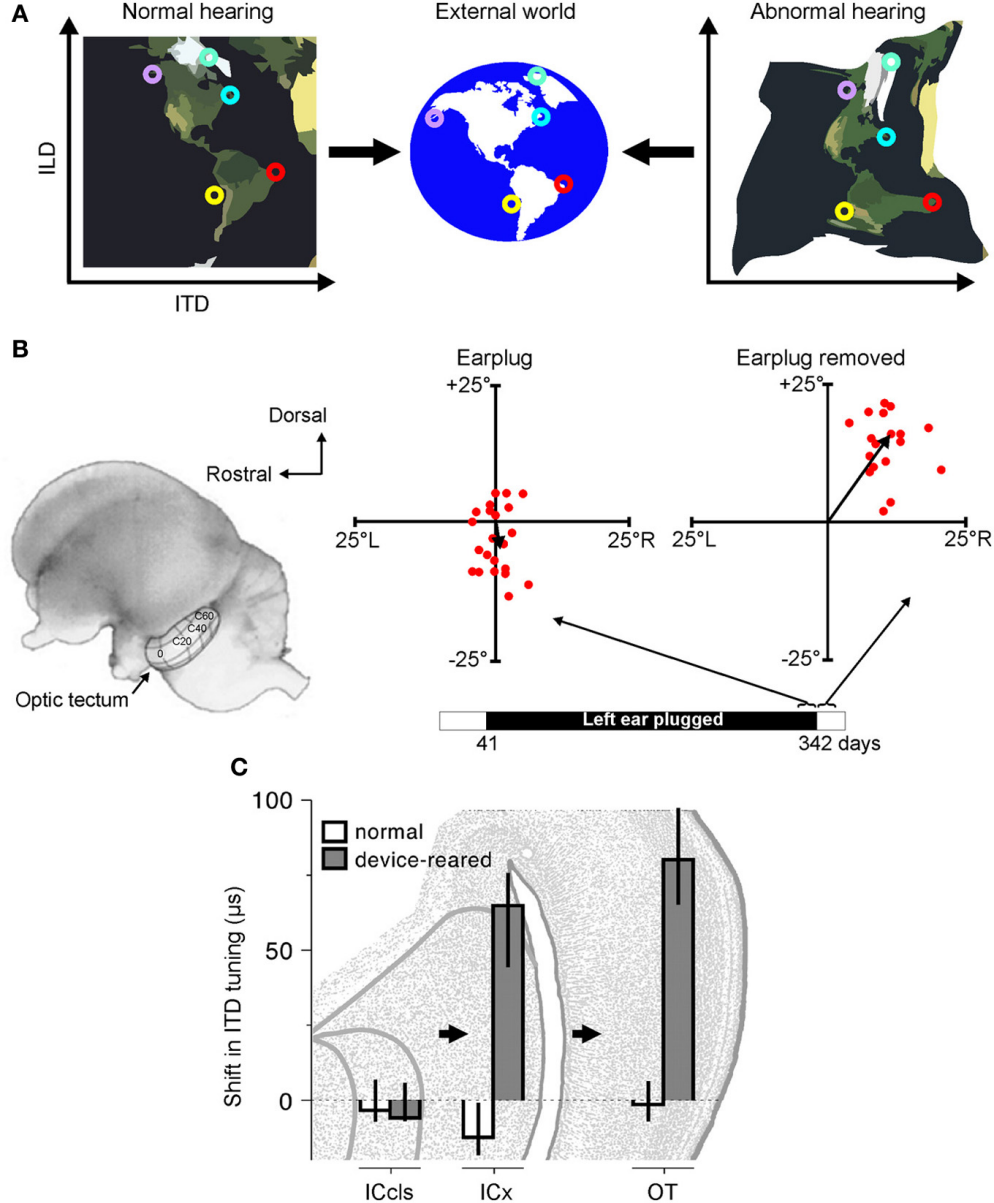
**Adaptation to abnormal cues can be achieved by remapping the relationship between cue values and sound-source location. (A)** Under normal listening conditions (left), specific combinations of cue values correspond to particular locations in the external world. Small circles of the same color represent particular cue combinations and their corresponding locations in the external world. Under abnormal listening conditions, such as when one ear is occluded by an earplug, these relationships are distorted and altered (right). In order to use these abnormal cues for accurate sound localization, the brain must therefore learn that the same locations now correspond to different cue combinations. **(B)** At present, robust neurophysiological evidence for cue remapping has only been observed in barn owls reared with one ear occluded. Electrophysiological recordings from neurons in the optic tectum of these animals show that compensatory shifts take place in the neurons' auditory spatial tuning. Tectal neurons respond most strongly to visual and auditory stimuli presented from overlapping locations, with their receptive fields arranged systematically to produce topographically-aligned maps of visual and auditory space (represented by the contour lines superimposed on the optic tectum in the picture of the owl's brain). Recordings from the rostral region of the tectum in an owl that was reared with the left ear occluded until 342 days after hatching revealed little difference between the visual and auditory receptive field centers when the earplug was still in place (“Earplug”). Misalignment in elevation is plotted on the ordinate, with misalignment in azimuth plotted on the abscissa. When the ear was occluded, the data points cluster around the origin, demonstrating that auditory and visual receptive fields are broadly in register. Following earplug removal, however, the receptive fields became systematically misaligned in both azimuth and elevation, indicating that the neurons were tuned to binaural cue values that no longer corresponded to their preferred visual location. **(C)** Site of auditory plasticity in the ascending auditory pathway of the barn owl. Frequency-dependent shifts in ITD tuning are plotted for barn owls reared either with normal hearing or with a passive filtering device in one ear that delays and attenuates sound. Positive values indicate shifts in ITD tuning that compensate for the effects of the device. Bars and lines show medians and interquartile ranges. Data are shown for the optic tectum (OT), external nucleus of the inferior colliculus (ICx) and the lateral shell of the central nucleus of the inferior colliculus (ICcls). Shifts in ITD tuning emerge at the level of the ICx. Modified with permission from Knudsen (1985) and Gold and Knudsen (2000b).

In principle, spatial hearing could adapt to a unilateral hearing loss in two distinct ways. First, in situations where the affected ear retains some acoustical sensitivity, the auditory system could utilize the cues that have been altered by hearing loss. In particular, this would require the brain to acquire new mappings between specific locations and the values of individual spatial cues (Figure 2A). In other words, the auditory system could adapt by learning to reinterpret the spatial meaning of particular acoustical inputs. Prior to the onset of hearing loss, for example, a sound located directly in front of an observer is likely to produce an ILD of zero, since the sound will be of equal intensity at each ear. However, if the sound transmitted to one ear is attenuated due to hearing loss, sounds located in front of the observer will no longer produce an ILD of zero. Instead, an ILD of zero may be produced by sounds originating from more peripheral locations ipsilateral to the hearing loss. In such circumstances, the auditory system could therefore adapt by adjusting the ILD sensitivity of neurons to compensate for the imbalance in inputs between the ears.

Thus, far, the clearest evidence for cue remapping has been obtained by studies of partial unilateral hearing loss in the developing barn owl. In particular, sound localization behavior in this species readily adapts to a unilateral hearing loss introduced early in development (Knudsen et al., 1984a). At a neural level, this adaptation is paralleled by shifts in ILD and ITD tuning, thereby changing the location of the receptive fields in ways that compensate for the effects of hearing loss (Figures 2B,C). For example, compensatory shifts in ITD sensitivity emerge at the level of ICx (Gold and Knudsen, 2000b) and, in turn, are observed in the optic tectum (Gold and Knudsen, 2001), thalamus (Miller and Knudsen, 2003) and forebrain (Miller and Knudsen, 2001). Similarly, adaptive shifts in ILD sensitivity initially appear in the brainstem nucleus that is the first site of ILD processing in the barn owl (Mogdans and Knudsen, 1994), prior to subsequent elaboration at the level of the ICx (Mogdans and Knudsen, 1993), optic tectum (Mogdans and Knudsen, 1992), and forebrain (Miller and Knudsen, 2001).

There are, however, key differences in the way in which barn owls and mammals localize sound, which likely reflect the independent evolution of mechanisms for sound localization in different groups of vertebrates (Grothe et al., 2010). Consequently, we should not necessarily expect the way the brain responds to unilateral hearing loss to be the same in birds and mammals. Indeed, in contrast to the experiments carried out in barn owls, very little evidence has been obtained for cue remapping in mammals. Thus, cats and rats reared with a partial unilateral hearing loss similar to that used in barn owl studies do not show compensatory shifts in the neural sensitivity to binaural spatial cues, either in the inferior colliculus (IC) (Clopton and Silverman, 1977; Silverman and Clopton, 1977; Moore and Irvine, 1981; Popescu and Polley, 2010) or the primary auditory cortex (Brugge et al., 1985; Popescu and Polley, 2010). Although it is conceivable that compensatory changes occur at higher levels of processing, these results highlight the possibility that cue remapping may not occur under all circumstances when a unilateral hearing loss is experienced.

Although there is currently very little evidence in mammals for experience–dependent adjustments in binaural cue sensitivity equivalent to those seen in barn owls, this does not necessarily mean that mammals are incapable of adapting in this manner to the altered cues produced by a unilateral hearing loss. In situations where normal hearing cannot be restored, it is therefore important to ask whether it might be possible to devise targeted intervention strategies that promote adaptive adjustments in ILD or ITD sensitivity. The mechanisms of plasticity revealed by research in barn owls therefore illustrate the potential for adaptive changes in the encoding of binaural spatial cues, even if the immediate clinical implications of this work remain unclear.

### Cue reweighting

A compensatory adjustment in neural sensitivity represents one viable mechanism for adapting to changes in auditory spatial cues, but this could also be achieved by the auditory system becoming more dependent on other cues that remain unchanged. In the specific case of unilateral hearing loss, this would require the auditory system to ignore the affected binaural spatial cues and instead rely more on the monaural spatial cues available to the intact ear. For individuals with a complete loss of hearing in one ear, this may be the only way in which a recovery in sound localization accuracy can be achieved. Indeed, behavioral evidence in at least some unilaterally deaf humans supports the idea that monaural spectral cues might be used to localize sounds in the horizontal plane (Slattery and Middlebrooks, 1994; Van Wanrooij and Van Opstal, 2004). Similarly, in cases of partial hearing loss, binaural cues are eliminated when the sound level is insufficiently high to be transmitted to the affected ear, which appears to enable humans to use spectral cues at these lower sound levels (Van Wanrooij and Van Opstal, 2007).

In such cases, however, it is unclear whether this amounts to cue reweighting, since these individuals do not have access to binaural spatial cues. Nevertheless, in situations where altered binaural cues remain available, it is clear that spatial hearing can adapt to a partial unilateral hearing loss by relying to a greater extent on the spectral cues provided to the intact ear. This has been demonstrated in both ferrets and humans with a unilateral hearing loss experienced either during adulthood (Kacelnik et al., 2006; Kumpik et al., 2010; Agterberg et al., 2012) or development (Newton, 1983; Keating et al., 2013a).

At a neuroanatomical level, developmental studies of unilateral hearing loss in mammals have demonstrated a relative weakening of the pathways that convey input from the affected ear, which include reduced connectivity, reductions in the size and number of neurons as well as changes in dendritic morphology (Tollin, 2010). Changes consistent with a weakening of these pathways have been observed in a variety of brain regions, including the cochlear nucleus (Coleman and O'Connor, 1979; Webster and Webster, 1979; Blatchley et al., 1983; Webster, 1983b; Moore and Kowalchuk, 1988), superior olive (Webster and Webster, 1979; Webster, 1983a; Sanes et al., 1992; Russell and Moore, 1999) and IC (Webster, 1983a,b), although the precise nature of these changes varies across different brain regions and different types of hearing loss. Studies of unilateral conductive hearing loss have shown that the neurophysiological representation of the developmentally occluded ear is similarly weakened, with neurons in the IC (Clopton and Silverman, 1977; Silverman and Clopton, 1977; Popescu and Polley, 2010) and auditory cortex (Brugge et al., 1985; Popescu and Polley, 2010) becoming relatively more driven by acoustical input provided to the intact ear. Although these studies were unable to measure the relative weight given to different spatial cues, a change in the relative efficacy with which each ear can activate central auditory neurons is precisely what would be expected if the auditory system were to become more dependent upon the monaural spatial cues provided by the intact ear.

**Figure 3:**
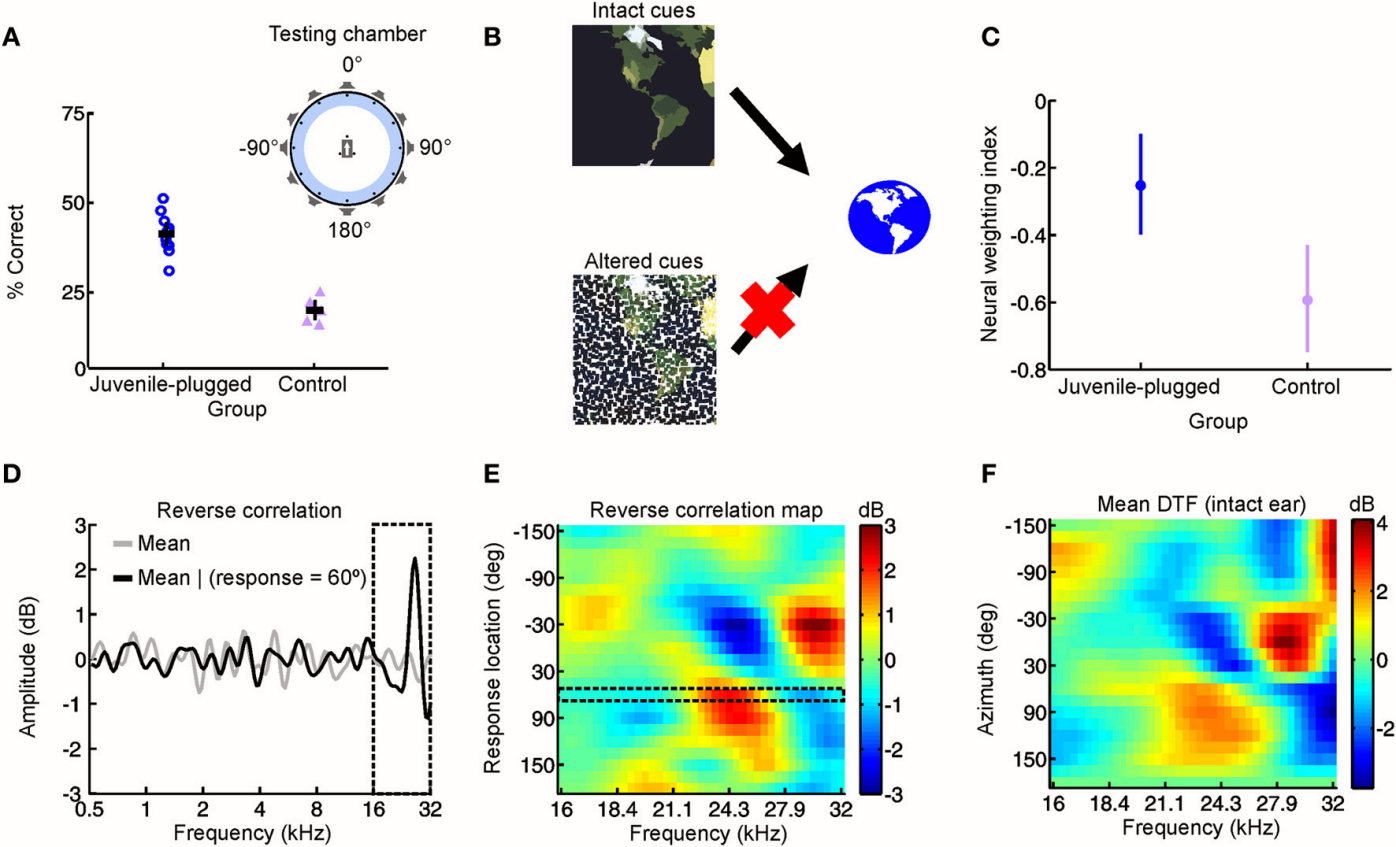
**Adapting to a unilateral hearing loss by changing the dependence of the auditory system on different spatial cues. (A)** Performance on an approach-to-target sound localization task is shown for normally-reared, control ferrets fitted with an earplug in one ear for the first time, as well as ferrets reared with a unilateral earplug (juvenile-plugged) and tested with an earplug in the developmentally-occluded ear. The animals initiated a trial by waiting on a central platform and approached the source of a sound presented from one of 12 loudspeakers positioned at equal intervals around the periphery, as illustrated in the accompanying schematic (top right). Juvenile-plugged animals performed the task with much greater accuracy than controls. **(B)** When spatial cues are altered or degraded by hearing loss, the auditory system can adapt by becoming less dependent on the abnormal cues and more dependent on the cues that remain intact. **(C)** Cue reweighting in primary auditory cortex. Neural weighting index is shown for neurons in the primary auditory cortex of juvenile-plugged ferrets while a virtual earplug was experienced in the developmentally-occluded ear. Stimuli were presented over earphones so that individual cues could be manipulated independently, which enabled a weighting index to be constructed. Higher values indicate that relatively more weight was given to the spatial cues provided by the intact ear. Data are also shown for normally-reared, control animals experiencing a virtual earplug in one ear. Neural weighting index values are higher in juvenile-plugged animals than controls, indicating greater reliance on the unchanged spectral cues provided by the intact ear. **(D–F)** Behavioral reweighting of auditory spatial cues revealed using reverse correlation. If approach to target localization responses are determined by the spectral cues provided by the intact ear, it is possible to recover these cues using reverse correlation. Juvenile-plugged ferrets performed the task in **(A)**, but the stimulus spectra were randomized across trials. The mean stimulus spectrum across all trials was very close to zero (gray line). However, on the subset of trials on which behavioral responses were made to a particular location (60° in the example shown), the mean stimulus spectrum deviated considerably from zero, with distinct spectral features emerging at frequencies >16 kHz **(D)**. Repeating this analysis for each response location produced a reverse correlation map **(E)**, which closely resembled the directional transfer function (DTF) of the intact (right) ear **(F)**. These results indicate that localization behavior in juvenile-plugged animals is guided by spectral features that resemble those produced by the directional filtering properties of the intact ear. This was not the case in controls, indicating that the juvenile-plugged animals had developed a greater dependence on, and therefore adapted to the unilateral hearing loss by giving greater weight to, the spectral cues that are unaffected by an earplug. Modified with permission from Keating et al. (2013a).

In a recent study, we therefore set out to test this cue reweighting hypothesis explicitly (Figure 3) (Keating et al., 2013a). We found that ferrets reared with a hearing loss in one ear were able to localize sounds accurately when tested as adults, despite wearing an earplug in the developmentally occluded ear (Figure 3A). Moreover, these behavioral experiments revealed that they did so by relying more on the monaural spectral cues provided to the intact ear (Figures 3D–F). At a neurophysiological level, this was paralleled by a corresponding reweighting of auditory spatial cues in the primary auditory cortex, with neurons carrying relatively less information about binaural spatial cues and relatively more information about the spectral cues that were unaffected by the hearing loss (Figure 3C). Thus, the animals were able to adapt to a unilateral hearing loss during the postnatal period when the auditory system is particularly plastic by giving greater weight to the spatial cues that remain unchanged. In conjunction with previous work in humans (Newton, 1983), these results therefore show that cue reweighting represents a viable strategy for adapting to developmental changes in sensory input.

### Choice of adaptive strategy

These studies demonstrate that the developing auditory system possesses the capacity to adapt to a partial unilateral hearing loss in one of two distinct ways. Whereas barn owls can adjust their sensitivity to the altered binaural cues (Keuroghlian and Knudsen, 2007) (Figure 2), mammals can accommodate the imbalance in inputs between the ears by becoming more dependent on the monaural spatial cues that remain intact (Newton, 1983; Keating et al., 2013a) (Figure 3). At present, however, it is unclear what determines which of these strategies is adopted. Perhaps the most obvious answer is that species differences may play an important role. Given that the barn owl possesses highly-specialized neural machinery for processing sound source location, the possibility of differences between species is certainly plausible.

The barn owl, for example, is capable of using fine-structure ITDs over the full range of frequencies to which it is sensitive, a truly remarkable feat that is made possible by phase-locking to much higher sound frequencies than is the case in mammals (Köppl, 1997). The acoustical properties of the head and ears also mean that this species respectively uses ITDs and ILDs for localizing sound sources in azimuth and elevation (Moiseff and Konishi, 1981; Moiseff, 1989). In this respect, barn owls differ considerably from mammals, which primarily use both ITDs and ILDs for determining the azimuth of a sound (Macpherson and Middlebrooks, 2002). Moreover, as discussed in an earlier section, there are differences in the way in which ITDs are encoded between these species (Harper and McAlpine, 2004). For these reasons, it is therefore possible that barn owls are capable of remapping binaural spatial cues onto abnormal spatial locations, whereas developing mammals are not.

On the other hand, barn owls may adapt to the altered binaural cues simply because this is the only viable strategy for adapting to a unilateral hearing loss in this species. In our experiments in ferrets that were monaurally deprived during development, we found that adaptive reweighting of auditory spatial cues is specific to the high sound frequencies (above approximately 16 kHz; Figure 3D) where spectral cues in this species are likely to be most informative (Keating et al., 2013a). Because their upper frequency limit of hearing is only approximately 10 kHz, it is possible that barn owls may not have access to the high-frequency spectral cues that would enable adaptation via cue reweighting, and may instead be forced to map altered binaural spatial cues onto their correct sound locations in order to adapt to a unilateral hearing loss. However, the frequency range over which the auditory periphery generates useful monaural spatial cues varies with the dimensions of the head and external ears. For example, studies in adult humans, where these structures are relatively large, have shown that subjects can learn to use abnormal spectral features available at much lower frequencies to make elevation judgments (Van Wanrooij and Van Opstal, 2005). Confirmation of whether barn owls are capable of experience-dependent cue reweighting will therefore require determining whether the spectral cues generated by the facial ruff in this species (Hausmann et al., 2009) contribute more to sound localization in birds that have adapted to a unilateral hearing loss.

Although these different viewpoints provide alternative, and perhaps complementary, explanations for the apparent differences between species, they remain speculative. As such, they serve to highlight important limitations in our understanding of the fundamental mechanisms underlying developmental plasticity. Under certain circumstances, for example, it is possible that both remapping and reweighting strategies could be utilized for adaptation in the same species. This would be consistent with the results of sound localization measurements in human listeners with acquired conductive hearing loss in one ear (Agterberg et al., 2012), although other work indicates that subjects with normal hearing can adapt to a temporary unilateral hearing loss without learning to use abnormal binaural spatial cues (Kumpik et al., 2010). Nevertheless, there is evidence that adult humans may be able to adapt to altered ILDs and ITDs following some acoustical manipulations (Javer and Schwarz, 1995; Shinn-Cunningham et al., 1998), so further research is needed, particularly at a neurophysiological level, to determine the relative contributions of spatial cue remapping and reweighting to the ability of the auditory system to accommodate the changes in input associated with hearing loss.

Factors likely to influence which adaptation strategy is used include the experience of the individual prior to the onset of hearing loss and the extent to which hearing is restored in the affected ear. It is conceivable, for example, that the ability to use binaural spatial cues, whether normal or abnormal, depends on normal binaural hearing during development (Seidl and Grothe, 2005; Grothe et al., 2010; Litovsky et al., 2010; Agterberg et al., 2012). Consequently, in the case of partial unilateral hearing loss, unravelling the factors that determine the nature of the adaptive mechanism represents a key goal for future work, with implications that are of interest from both a clinical and fundamental perspective.

### Neural basis of cue reweighting

**Figure 4:**
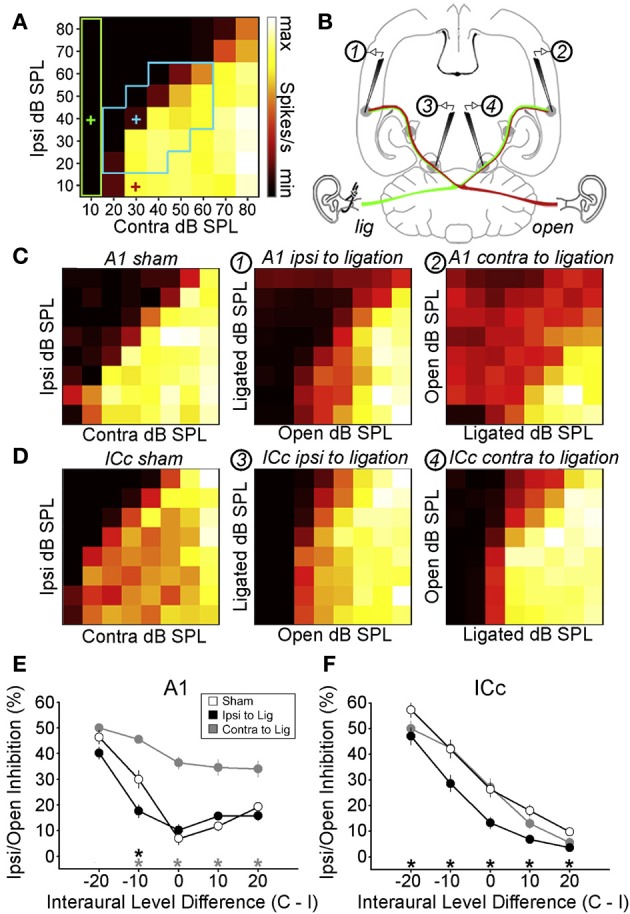
**Maladaptive effects of asymmetric hearing loss during development. (A)** Example of a binaural interaction matrix recorded from a unit in the central nucleus of the inferior colliculus (ICc) in a normally reared rat. Contralateral sound level is plotted against ipsilateral level, with color denoting the number of spikes fired for each combination. Firing rates typically increase as the contralateral level is increased, but are suppressed when the ipsilateral level exceeds that in the contralateral ear. For each interaural level combination enclosed by the blue box, binaural suppression was quantified by comparing it with the linear sum of its monaural intercepts (e.g., blue cross relative to the sum of the red and green crosses). **(B)** To investigate the developmental effects of monaural deprivation, rats were reared with a hearing loss in one ear that was induced by ligation of the ear canal, which was reversed prior to electrophysiological experiments. Bilateral recordings were then performed in the ICc and primary auditory cortex (A1) of these animals and compared with data from sham-operated controls reared with normal hearing. **(C,D)** Examples of binaural interaction matrices from A1 **(C)** and ICc **(D)** in sham operated controls (left), and in ligated animals. For ligated animals, data are shown for the hemisphere ipsilateral (middle) and contralateral (right) to the ligated ear. Color scales and axis labels are identical to (A). **(E,F)** Ipsilaterally mediated suppression expressed as a function of ILD for A1 **(E)** and ICc **(F)** recordings. Data are shown for sham operated controls (open symbols) as well as ligated animals, both contralateral (gray) and ipsilateral (black) to the ligated ear. Asterisks denote significant differences between ligated animals and controls, with asterisk grayscale indicating the hemisphere in which the comparison was made. Error bars show SEMs. Ipsilaterally mediated suppression in A1 of monaurally deprived animals is increased in the hemisphere contralateral to the deprived ear **(E)**, but not in the corresponding hemisphere of the ICc **(F)**. Conversely, ipsilaterally mediated suppression is reduced in the ICc ipsilateral to the deprived ear **(F)**, but this effect is not apparent at the level of A1 **(E)**. These results suggest that monaural deprivation induces persistent changes in the strength of ipsilateral input, which acts to weaken the representation of the deprived ear at the level of the ICc and strengthen the representation of the intact ear at the level of A1. In both cases, this increases the relative strength of the intact ear, and produces maladaptive shifts in ILD sensitivity. Modified with permission from Popescu and Polley (2010).

As previously discussed, the capacity of ferrets to adapt to a conductive hearing loss in one ear during postnatal development relies on their greater use of monaural spectral cues provided by the contralateral ear, with equivalent cue reweighting being displayed by auditory cortical neurons in these animals (Keating et al., 2013a). It is presently unclear, however, whether this reflects plasticity at the level of the cortex itself or at lower levels of processing. In mammals, for example, it is known that a unilateral hearing loss produces changes in the binaural tuning properties of IC neurons (Clopton and Silverman, 1977; Silverman and Clopton, 1977; Moore and Irvine, 1981; Popescu and Polley, 2010) (Figures 4D,F). Since IC neurons can combine the information provided by different auditory spatial cues (Chase and Young, 2005), it is possible that changes in cue integration are implemented at the level of the IC. More generally, this is consistent with the idea that the IC may be an important site of plasticity due to its role as a major site of convergence for many auditory pathways (Yu et al., 2007).

On the other hand, some measures of spatial processing by IC neurons are immune to the effects of a unilateral hearing loss, despite undergoing changes at higher levels of processing (Popescu and Polley, 2010) (Figures 4C,E). Moreover, plasticity observed in the spatial response properties of IC neurons is often more extensive at higher levels of processing (Popescu and Polley, 2010). This suggests that additional changes may emerge either in the thalamus or the cortex (Popescu and Polley, 2010; Polley et al., 2013). Indeed, the involvement of multiple processing levels in adaptive plasticity has been demonstrated in adult ferrets. Thus, the ability of these animals to relearn to localize sound following a unilateral hearing loss depends on corticocollicular connections (Bajo et al., 2010) as well as the integrity of the cortex (Nodal et al., 2010). Similarly, plasticity induced by conditioning or by focal electrical stimulation in the tonotopic representation of the auditory cortex leads to changes in frequency tuning in the IC that depend on the relationship between activity in these two structures (Yan et al., 2005). There is therefore growing evidence that the effects of sensory experience on auditory perception may be mediated at the level of the cortex and then transmitted to subcortical sites via descending feedback connections (Bajo and King, 2013) (Figure 1C).

Whilst it is unclear whether similar principles apply to developing animals, these studies highlight the possibility that cue reweighting may be implemented by interactions between the midbrain and cortex. Consistent with this view, multisensory cue integration in the SC is thought to depend on descending input from “association” cortex, both during adulthood and development (Wallace and Stein, 1994; Jiang et al., 2006). However, since these results do not necessarily imply that cortical input influences unisensory integration at a subcortical level (Alvarado et al., 2007), a key step toward testing this model would be to determine whether IC neurons show evidence for cue reweighting.

### Incomplete and maladaptive changes in spatial processing

Although developmental plasticity can be beneficial, enabling an individual to adapt to a particular environment, these adaptive changes are often incomplete. For example, ferrets reared with an earplug in one ear localize sounds less well whilst wearing an earplug than controls with normal hearing (Keating et al., 2013a). Similarly, children who experience a unilateral hearing loss appear to be unable to fully adapt to the abnormal acoustical input available to them (Newton, 1983), and show clear deficits in sound localization (Viehweg and Campbell, 1960; Humes et al., 1980; Newton, 1983; Bess et al., 1986). In addition, whilst barn owls show very little residual bias in sound localization following adaptation to a unilateral hearing loss, the spatial precision of behavioral responses often remains slightly worse than controls (Knudsen et al., 1984a). Thus, despite clear differences between birds and mammals, an inability to fully compensate for the effects of asymmetric hearing loss is common to many different species. For tasks that are likely to require more complex processing, such as binaural unmasking in the presence of spatially discordant signals and noise, there is currently little evidence for any adaptation (Moore et al., 1999). The degree to which the developing auditory system can accommodate changes in input following hearing loss therefore depends on the nature of the task, and even where adaptation clearly occurs, such as in directional hearing tasks, this may not be enough to fully restore normal performance levels.

Perhaps more damagingly, however, any adaptive changes that do occur can become maladaptive if the environment is subsequently altered. This is particularly relevant to situations where a developmental hearing loss is resolved later in life, either spontaneously or through clinical intervention. In such cases, the auditory system may have altered the way in which spatial cues are processed, thereby compromising its ability to take full advantage of normal hearing when it becomes available.

Following a developmental hearing loss in one ear, for example, barn owls are, at least initially, unable to localize sounds correctly when normal hearing is restored (Knudsen et al., 1984b). This is because these animals have adapted to the imbalance in input between the two ears by remapping binaural spatial cues onto abnormal spatial locations. Although these mappings permit accurate localization when a hearing loss is experienced in one ear, they are not appropriate for localization under normal hearing conditions. The animals therefore exhibit systematic errors in sound localization when normal hearing becomes available, which can persist indefinitely if hearing is not restored early enough in development (Knudsen et al., 1984b). At a neural level, this is paralleled by persistent abnormalities in the tuning to binaural spatial cues (Keuroghlian and Knudsen, 2007) (Figures 2B,C).

Similarly, in mammals reared with a stable hearing loss in one ear, the neural representation of the developmentally occluded ear is typically [though not always (Moore and Irvine, 1981)] weakened, and does not immediately return to normal when the cause of the hearing loss is removed (Clopton and Silverman, 1977; Silverman and Clopton, 1977; Brugge et al., 1985; Popescu and Polley, 2010) (Figure 4), even when the period of monaural hearing loss is relatively brief (Polley et al., 2013). This is consistent with the cue reweighting mechanism of adaptation, which involves relying more on the input provided by the intact ear and less on the input from the occluded ear. Although this plasticity may help the auditory system to adapt to a hearing loss in one ear, it has been proposed that it may also be responsible for amblyaudia (Popescu and Polley, 2010; Whitton and Polley, 2011; Polley et al., 2013), a persistent condition in which the brain is unable to fully exploit the acoustical input provided to one ear. This is because the changes associated with asymmetric hearing loss can impair binaural processing of normal acoustical inputs, producing deficits in ILD sensitivity when normal hearing is restored (Moore and Irvine, 1981; Popescu and Polley, 2010; Polley et al., 2013).

In a recent study, for example, rats were reared with a conductive hearing loss in one ear and binaural interactions were quantified in the ICc and auditory cortex after the hearing loss was reversed (Popescu and Polley, 2010) (Figures 4A,B). In the ICc ipsilateral to the developmentally deprived ear, ipsilaterally-mediated suppression was weakened (Figures 4D,F), but this effect was not apparent at the level of A1 (Figures 4C,E). Conversely, ipsilaterally-mediated suppression in A1 of monaurally deprived animals was increased in the hemisphere contralateral to the deprived ear (Figures 4C,E), but not in the corresponding hemisphere of the ICc (Figures 4D,F). Together, these results show that binaural interactions may be altered by a developmental hearing loss in one ear, which can subsequently impair processing when normal hearing is restored.

Consistent with this view, humans and guinea pigs with a developmental history of asymmetric hearing loss tend to show persistent deficits in behavioral measures of binaural and spatial hearing even after normal hearing is restored (Clements and Kelly, 1978; Beggs and Foreman, 1980; Hall and Derlacki, 1988; Pillsbury et al., 1991; Wilmington et al., 1994; Moore et al., 1999; Gray et al., 2009). Since spatial hearing is important for listening in noisy and reverberant environments (Yost, 1997), this can affect performance in social and educational settings. In this way, deficits in spatial hearing may impair or delay linguistic, cognitive and social development (Moore et al., 2003; Tollin, 2010; Whitton and Polley, 2011).

Nevertheless, whilst long periods of unilateral hearing loss may weaken the neural representation of the deprived ear, this representation does not appear to be eliminated entirely. For example, ferrets reared with an earplug in one ear eventually show normal levels of binaural unmasking, although this can take up to approximately 2 years after the cause of the hearing loss has been removed (Moore et al., 1999). Consistent with these results, bilaterally deaf individuals who receive a unilateral cochlear implant typically retain some sensitivity to the non-implanted ear when a second implant is received in that ear later in life, despite experiencing a long period of unilateral stimulation (Kral et al., 2013). Although generally inferior to that associated with the first implant, this residual sensitivity might provide a neural basis for rehabilitation. A key goal for future research will therefore be to determine whether recovery from hearing loss during development can be enhanced or accelerated by specific training regimens, an approach that has been successfully used to treat deficits in language comprehension (Merzenich et al., 1996; Tallal et al., 1996). In this respect, it is critical to identify individuals who are particularly at risk and determine the developmental time point at which intervention is likely to be most successful.

## Effects of asymmetric hearing loss throughout the lifespan

### Differences between developmental and adult plasticity

One of the key findings to emerge from studies of spatial hearing following unilateral hearing loss is that the timing of hearing loss plays a critical role in mediating its effects. Early work in the barn owl, for example, emphasized the notion of sensitive or critical periods (Knudsen et al., 1984a,b), suggesting that auditory representations of space become relatively fixed at a particular stage of development. Initially, it was thought that the critical period for spatial hearing was determined by age rather than sensory experience (Knudsen and Knudsen, 1986). Subsequent work, however, suggested that critical periods for the visual recalibration of spatial hearing may be extended by environmental enrichment (Brainard and Knudsen, 1998). In a similar vein, the critical period for the effects of auditory experience on the organization of frequency maps in the auditory cortex (Zhang et al., 2001; De Villers-Sidani et al., 2007) can be extended by exposure to environmental noise (Chang and Merzenich, 2003).

A number of studies in which individuals have been exposed to altered auditory or visual experience have, however, demonstrated that spatial hearing remains plastic in adulthood [reviewed by Irvine and Wright (2005), Keuroghlian and Knudsen (2007); King (2009); King et al. (2011)]. In particular, adaptive changes in sound localization have been observed in adult ferrets (Kacelnik et al., 2006; Nodal et al., 2010; Irving et al., 2011) and humans (Kumpik et al., 2010; Irving and Moore, 2011; Strelnikov et al., 2011) after introducing an asymmetric hearing loss. While there are clear similarities with the experience-dependent changes that can take place during development, with adaptation in mammals likely to involve cue reweighting irrespective of age (Kacelnik et al., 2006; Kumpik et al., 2010; Keating et al., 2013a), this does not mean that the neurophysiological basis for this is necessarily the same. Indeed, plasticity in the young brain may differ from that in the adult in a number of important ways. Adult plasticity, for example, seems to depend to a greater extent on behavioral training (Bergan et al., 2005; Kacelnik et al., 2006), which may be mediated by increased focus and heightened arousal (Keuroghlian and Knudsen, 2007). In this respect, the plasticity of spatial hearing in adults parallels that of frequency and intensity processing (Polley et al., 2006).

Additional work, however, suggests that adult plasticity is possible even in the absence of behavioral training, with changes in frequency tuning induced by passive exposure to specific acoustic environments (Noreña et al., 2006; Zhou et al., 2008; Pienkowski and Eggermont, 2009, 2010; De Villers-Sidani and Merzenich, 2011; Pienkowski et al., 2011; Zhou et al., 2011; Pienkowski and Eggermont, 2012; Zheng, 2012; Zhou and Merzenich, 2012; Pienkowski et al., 2013). The results of one particular study, for example, suggest that it may be possible to reinstate more extensive plasticity in adults by exposure to moderate levels of acoustic noise (Zhou et al., 2011), a result that parallels findings of increased plasticity in visual cortex following a period of dark exposure (Duffy and Mitchell, 2013). This suggests that patterned sensory input may affect not only the development of neural processing but its subsequent maintenance later in life (Shepard et al., 2012).

On the other hand, the effects of attention and reward are not limited to adults, with developing circuits relevant to vocalization showing a similar susceptibility to these influences (Doupe and Kuhl, 1999). Together, these results question whether behavioral training plays a unique role in adult plasticity, and instead point toward the possibility that training enhances and accelerates plasticity throughout the lifespan. Indeed juvenile animals may be even more susceptible to the effects of auditory training than adults. For example, Sarro and Sanes (2011) found that behavioral training induced greater plasticity in juvenile gerbils than an equivalent amount of training in adulthood. The key difference between juveniles and adults may therefore lie in their susceptibility to change, with developing animals capable of more rapid and extensive changes. The reasons for this difference remain to be identified, but a variety of factors are thought to contribute, including differences in neuromodulatory influences (Shepard et al., 2012) as well as the relative balance of excitation and inhibition (Dorrn et al., 2010).

### Importance of early developmental experience

Although the auditory system can adapt to a unilateral hearing loss both during development and in adulthood, the consequences of this form of auditory deprivation have been shown to vary depending on the point in development at which hearing loss first occurs. Earlier developmental onsets result in a greater weakening of the neurophysiological (Clopton and Silverman, 1977; Popescu and Polley, 2010) and neuroanatomical (Blatchley et al., 1983; Webster, 1983b) representations corresponding to the affected ear. This suggests that neural circuits responsible for integrating inputs from the two ears may be particularly labile early in development.

If spatial hearing were particularly vulnerable to hearing loss early in development, we might expect clinical intervention to be more successful early in life. Consistent with this view, one recent study used hearing aids to restore balanced hearing to children who had previously been diagnosed with a sensorineural hearing loss in one ear (Johnstone et al., 2010). When tested more than 1 year later, children who received a hearing aid before the age of 5 showed improved sound localization performance under aided hearing conditions, but children who received a hearing aid later in life did not. Although the authors of this study noted that the two groups were tested at different ages, which could have influenced the outcome, these results are consistent with the notion that early developmental experience is particularly important. Further evidence for this view has been observed in studies of patients fitted with bone conduction devices following either congenital or acquired asymmetric hearing loss. In particular, patients with a congenital hearing loss benefit less from wearing a bone conduction device than patients who had normal hearing during early development (Agterberg et al., 2011).

Recipients of cochlear implants who are congenitally deaf also provide a unique opportunity to study this question. In recent work, for example, sequential implantation of the two ears was found to impair the ability to make use of the second implant if it was implanted much later in life than the first (Graham et al., 2009; Gordon et al., 2013; Illg et al., 2013), with the associated neural impairments persisting for at least 3–4 years following the onset of bilateral stimulation. Similarly, unilateral cochlear implantation in congenitally deaf cats leads to greater functional dominance of the implanted ear, but only if animals experience unilateral stimulation early in life (Kral et al., 2013). This suggests that the auditory system may be particularly vulnerable to early experience of unilateral stimulation, which has profound implications for rehabilitation strategies following asymmetric hearing loss.

**Figure 5:**
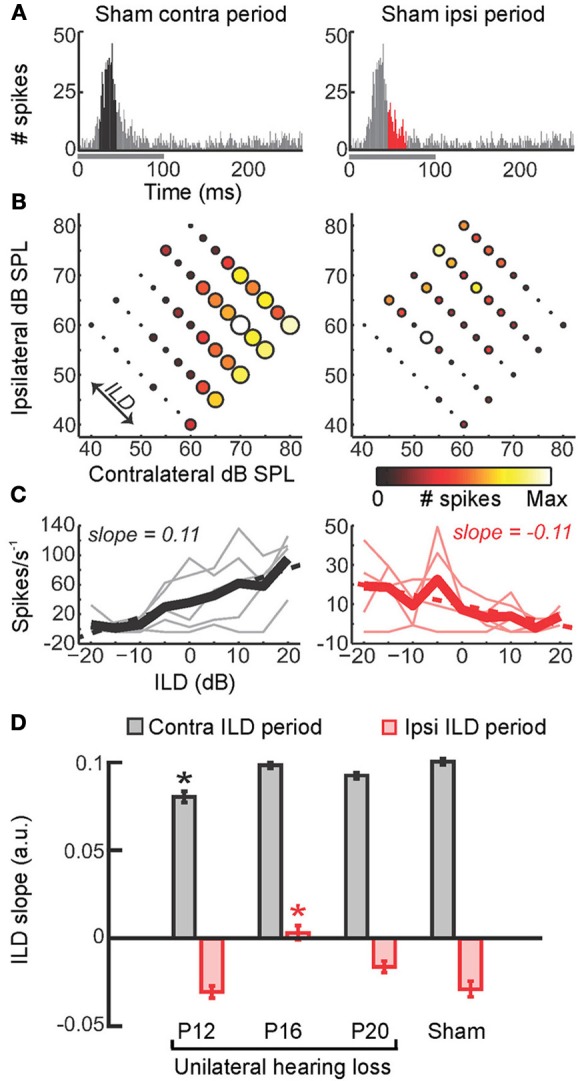
**Precise timing of unilateral hearing loss influences developmental outcome. (A)** Poststimulus time histograms (PSTHs) of spikes recorded from neurons in the primary auditory cortex of the mouse can be divided into windows that show the strongest differential sensitivity to contralateral (black, left) and ipsilateral (red, right) ILDs. Greater contralateral ILD sensitivity is typically a feature of short-latency responses, whereas greater sensitivity to ipsilateral ILDs tends to be seen in longer latency responses. **(B)** Firing rate as a function of ILD at 5 different average binaural levels, with each plot corresponding to the response window shown immediately above. Heat map is scaled to the normalized firing rate within each time window, whereas circle diameter is normalized to the maximum firing rate across both time windows. **(C)** Faint lines show firing rates as a function of ILD for each of the average binaural levels shown in **(B)**. Thicker lines show the average of ILD functions obtained with different average binaural levels. **(D)** Slopes of linear fits were calculated for the thick lines in **(C)** and provide a measure of ILD sensitivity, with larger slope values indicating greater sensitivity. Mean slope values (±s.e.m.) are shown for sham operated controls as well as mice that experienced brief periods (1–2 weeks) of unilateral hearing loss beginning at different ages (either postnatal day 12, 16, or 20). Contralateral and ipsilateral ILD sensitivity are both reduced by asymmetric hearing loss, but these different aspects of spatial processing are vulnerable at different stages of development. Asterisk indicates significant differences relative to (sham-operated) controls (*post-hoc* tests following ANOVA, *P* < 0.05). Modified with permission from Polley et al. (2013).

More recent work, however, points toward a more complex situation, in which different aspects of binaural sensitivity are vulnerable to hearing loss at different stages of development (Polley et al., 2013) (Figure 5). In particular, this study showed that cortical neurons tend to preferentially respond to contralateral locations with short-latency spikes and ipsilateral locations with longer latency spikes (Figures 5A–C). Hearing loss at specific ages can selectively impair one of these measures of binaural processing whilst leaving the other largely intact (Figure 5D). These results therefore point toward the possibility that vulnerability may be most pronounced when the neural circuits in question are maturing. Because different circuits are likely to mature at different times, this means that the consequences of hearing loss may depend on the precise developmental stage at which it is experienced.

## Effects of a recurring asymmetric hearing loss during development

### Intermittent periods of normal experience may reverse amblyaudia

**Figure 6:**
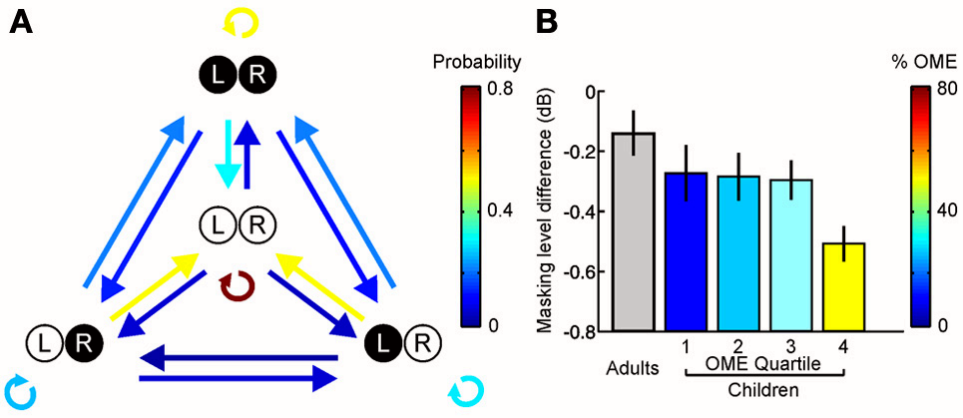
**Otitis media with effusion (OME) and its effects in humans. (A)** State transition matrix for different effusion states, obtained via a prospective study among a group of 95 children aged 0–3 years. Arrow color shows the probability of transitioning from one effusion state to another. Effusion was either present (filled circles) or absent (open circles) in the left (L) and right (R) ears. Individuals often alternate between periods of normal and abnormal hearing. **(B)** Masking level difference, which provides a measure of spatial listening in noisy environments, is shown for adults as well as children with a history of OME (mean ± s.e.m.). Children are divided into quartiles depending on the amount of OME experienced during the first 5 years of life, with the colors indicating the mean prevalence of OME in each quartile. Adults with unknown OME experience are shown in gray. A deficit in masking level difference was observed in the upper quartile of children that experienced OME, corresponding to children that experienced OME at least 50% of the time. Based on Hogan et al. (1997) and Hogan and Moore (2003).

Although the timing and duration of hearing loss are likely to have a significant impact on spatial hearing, the temporal pattern of the impairment may also be a critical factor. In this respect, most research in this area has been restricted to studying a particular type of auditory deprivation, namely a single episode of unilateral hearing loss that remains stable over time (Clopton and Silverman, 1977; Silverman and Clopton, 1977; Clements and Kelly, 1978; Moore and Irvine, 1981; Blatchley et al., 1983; Webster, 1983b; Brugge et al., 1985; Popescu and Polley, 2010; Polley et al., 2013). It is much less clear, however, what would happen if the auditory system were exposed to a recurring hearing loss in one ear separated by periods of normal hearing. From a clinical perspective, this is important because recurring periods of hearing loss are extremely common during infancy (Hogan et al., 1997; Whitton and Polley, 2011). Otitis media with effusion, for example, is typically experienced in discrete episodes separated by periods of normal hearing (Figure 6A). It is therefore of considerable interest to determine the impact of intermittent hearing loss on auditory development.

On the one hand, we might expect that the effects of hearing loss would be reduced by periods of normal hearing. Studies of the visual system, for example, have shown that the negative effects of occluding one eye during development can be at least partially (Mitchell et al., 2003, 2006, 2011), albeit not necessarily completely (Mitchell et al., 2009), reversed by providing cats with brief intermittent periods of normal visual experience. If the same were true of the auditory system, then the symptoms associated with amblyaudia might be similarly ameliorated by providing intermittent experience of normal hearing throughout development.

Alternatively, intermittent periods of hearing loss might be damaging, producing an unstable acoustical environment in which the auditory system is unable to develop properly. In one early study, for example, barn owls were reared with unilateral earplugs that alternated between the two ears every second day (Knudsen and Knudsen, 1986). In contrast to the effects of chronic occlusion of one ear, these animals failed to adapt to a hearing loss in either ear and therefore made large localization errors, suggesting that inputs need to remain stable for a minimum period of time for adaptive changes in spatial cue processing to take place.

**Figure 7:**
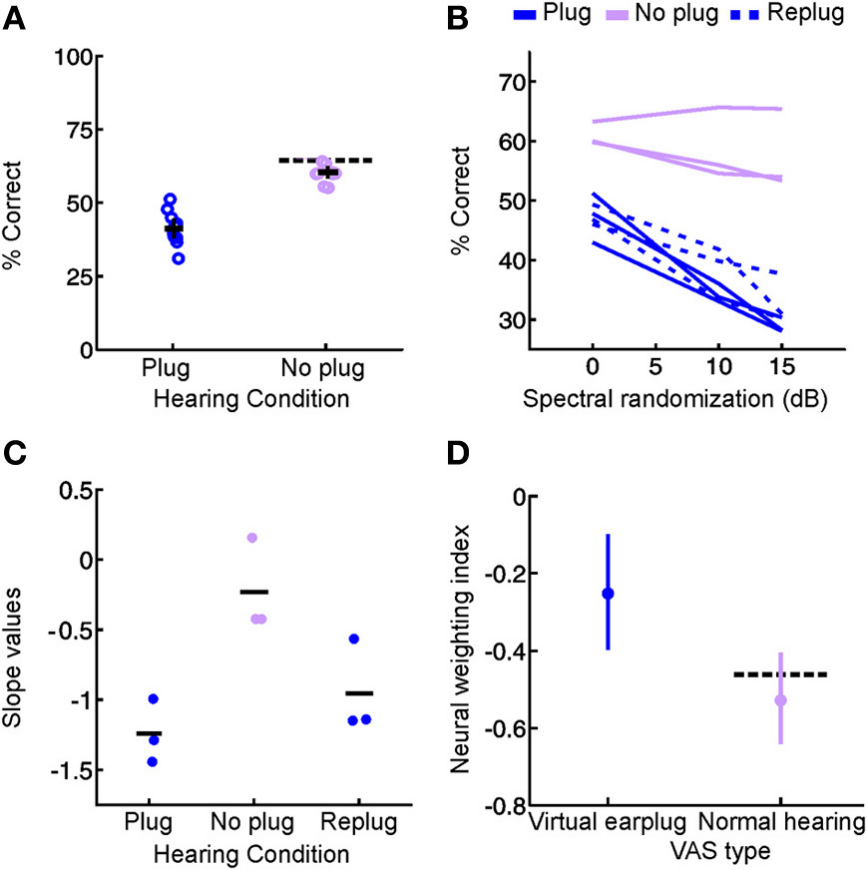
**Context-dependent reweighting of auditory spatial cues following a recurring developmental hearing loss in one ear. (A)** Sound localization performance of ferrets reared with an earplug in one ear, either in the presence or absence of an earplug. Each symbol represents data from an individual animal. Although juvenile-plugged ferrets adapt to an earplug (see Figure 3), their performance improves when the earplug is removed, and approaches the mean performance level of controls under normal listening conditions (dotted black line). This means that juvenile-plugged ferrets adapt to an asymmetric hearing loss without compromising their ability to localize accurately when normal hearing becomes available. Error bars denote bootstrapped 95% confidence intervals, with solid black lines showing group means. **(B)** Context-specific reweighting of auditory spatial cues. Randomizing stimulus spectra across trials is known to degrade the usefulness of spectral cues, since it becomes unclear whether spectral features arise from the filtering effects of the head and ears or are instead properties of the stimulus itself. Whilst wearing an earplug, sound localization performance in juvenile-plugged ferrets declined as the amount of spectral randomization was increased, but this effect largely disappeared once the earplug was removed. Each line shows data for an individual animal, either with an earplug in place (solid, dark blue), following earplug removal (solid, pink), or after the reintroduction of an earplug (dotted, dark blue). **(C)** To quantify the effects of spectral randomization, slope values were calculated for the lines in **(B)** and are plotted for different hearing conditions. Each symbol shows data from an individual animal, with solid black lines indicating group means. Performance of juvenile-plugged ferrets was only impaired by randomization (negative slope values) when one ear was occluded. This means that the localization behavior of juvenile-plugged ferrets became more dependent on the spectral cues available to the contralateral ear whenever a unilateral earplug was present. The lack of effect of spectral randomization in the absence of the earplug suggests that the animals were relying on binaural cues when normal inputs were available. **(D)** Neural weighting index values are shown for neurons in the primary auditory cortex of juvenile-plugged ferrets, either in the presence or absence of a virtual earplug. Higher values indicate greater reliance on the spectral cues provided by the developmentally non-occluded ear. In juvenile-plugged ferrets, neural weighting index values change depending on whether a virtual earplug is present or not. Controls do not show the same effect, and are indistinguishable from juvenile-plugged ferrets under normal hearing conditions (dotted black line shows mean neural weighting index values for controls). This means that recurring monaural deprivation during development leads to cortical neurons weighting auditory spatial cues differently depending on whether a hearing loss is experienced, providing a possible neural basis for the cue reweighting observed behaviorally. Modified with permission from Keating et al. (2013a).

We addressed this issue in our recent study in which ferrets were reared with a unilateral hearing loss by providing brief intermittent periods of normal hearing throughout development (Keating et al., 2013a). As shown in Figure 3, the sound localization behavior of these animals indicated that significant adaptation to the unilateral hearing loss had taken place. Instead of making systematic errors when normal inputs were provided in both ears, they were indistinguishable from controls in their ability to localize sounds (Figure 7A). Thus, although the animals showed clear evidence for experience-dependent changes in cue integration, these changes were selectively expressed, both behaviorally (Figures 7B,C) and neurophysiologically (Figure 7D), in the presence of a unilateral hearing loss, and disappeared whenever balanced binaural hearing was available (Figures 7B–D). These results contrast dramatically with the persistent changes observed following removal of the ear canal occlusion in animals reared with a stable hearing loss in one ear (Clopton and Silverman, 1977; Silverman and Clopton, 1977; Clements and Kelly, 1978; Moore and Irvine, 1981; Knudsen et al., 1984a; Brugge et al., 1985; Gold and Knudsen, 2000a; Popescu and Polley, 2010; Polley et al., 2013), which suggests that relatively brief periods of normal hearing may be able to preserve the ability to use binaural cues correctly.

This is consistent with clinical observations in humans, which show that the negative effects of a recurring hearing loss are reduced as the cumulative amount of abnormal hearing is decreased (Hogan and Moore, 2003) (Figure 6B). Together, these studies indicate that the symptoms of amblyaudia may be at least partially reversed by providing the developing auditory system with brief periods of normal hearing. Although it is currently unclear whether intermittent experience of normal hearing preserves the integrity of neuroanatomical pathways that are degraded by exposure to a stable unilateral hearing loss (Blatchley et al., 1983; Webster, 1983b), this is likely to be the case, and therefore remains an important question for future research.

Another important issue, however, concerns the amount of normal sensory experience that is required to avoid the negative effects of hearing loss. In humans, deficits in spatial hearing are observed if the proportion of abnormal hearing exceeds 50% (Hogan and Moore, 2003) (Figure 6B). In ferrets, however, normal sound localization abilities can develop if the proportion of normal hearing is approximately 20% (Keating et al., 2013a). Although there are many factors that could explain this difference, it is likely that different tasks may be more or less sensitive to abnormal hearing during development. Consequently, whilst relatively simple tasks, such as localizing single sound sources in quiet environments (Keating et al., 2013a), may require very little experience of normal hearing, performance of tasks that involve more complex processing (Hogan and Moore, 2003) may require relatively more experience of normal hearing for these abilities to be preserved following a period of asymmetric hearing loss (Wilmington et al., 1994). An additional possibility is that it may be beneficial to have access to at least one set of cues that remains stable over time. If this were the case, then this would explain why subjects reared with entirely normal hearing in one ear (Keating et al., 2013a) appear to be less affected by hearing loss than individuals who intermittently experienced abnormal hearing in either ear (Knudsen and Knudsen, 1986; Hogan and Moore, 2003). Future work, however, will be necessary to resolve this issue.

### Context-specificity of developmental plasticity

Although studies of recurring hearing loss are directly relevant to otitis media with effusion and its treatment (Hogan et al., 1997; Whitton and Polley, 2011), they also have broader implications for our understanding of sensory processing in complex multi-context environments (Qian et al., 2012). In naturalistic situations, for example, the sensory environment may change dramatically over time. This variability, however, may not be entirely random, but may instead have specific statistical properties. The acoustical properties of a classroom, for example, may be very different from those experienced beside a busy road, although each may remain similar over time. The acoustical environment may therefore transition between distinct contexts, each of which is characterized by relatively stable statistical properties (Qian et al., 2012). By alternating between normal and abnormal acoustical contexts, recurring forms of hearing loss therefore provide an excellent experimental model for studying a much wider class of problem faced by the brain.

Our finding that juvenile ferrets can adapt to a recurring hearing loss in one ear by relying more on the monaural spectral cues provided to the intact ear when the hearing loss is present, whilst maintaining sensitivity to binaural cues under normal hearing conditions, suggests that the developing auditory system can process spatial cues in different ways depending on the specific environmental context in which they are experienced (Keating et al., 2013a) (Figure 7). Such context-dependent processing therefore enables the auditory system to adapt to a unilateral hearing loss without compromising its ability to use normal spatial cues. On the grounds that bilingual individuals can learn to interpret the same acoustic tokens in different ways depending on the linguistic context in which they occur (Werker, 2012; Buchweitz and Prat, 2013), we can think of this type of plasticity as “spatial bilingualism.”

By showing that developmental plasticity enables context-dependent cue integration, this result ties together two distinct lines of research on cue integration. On the one hand, developmental studies have demonstrated that the emergence of cue integration depends on prior sensory experience (Wallace and Stein, 2007; Xu et al., 2012; Yu et al., 2013). On the other hand, numerous studies in adults have shown that cue weights can be rapidly updated to reflect their relative reliability (Ernst and Banks, 2002; Alais and Burr, 2004). In particular, behavioral experiments in adult humans have demonstrated that cue weights can be selectively updated for certain contexts or object classes, but not others (Jacobs and Fine, 1999; Atkins et al., 2001; Seydell et al., 2010). Very little is known, however, about the neural mechanisms that might enable the developing brain to learn to weight different cues (Fiser et al., 2010; Berkes et al., 2011).

Similarly, in the auditory system, there is accumulating neurophysiological evidence for various forms of context-dependent processing. Cortical activity, for example, shows rapid changes when individuals are required to perform specific tasks, with the nature of these changes being determined by the specific task requirements (Fritz et al., 2003, 2005; David et al., 2012; Mesgarani and Chang, 2012). Although these effects are thought to be mediated by attention, sensory context is also known to influence auditory processing, since the tuning properties of neurons can be modified by prior acoustical stimulation (Dean et al., 2008; Dahmen et al., 2010; Wen et al., 2012; Yaron et al., 2012; Nelken and De Cheveigne, 2013; Stange et al., 2013). Some studies have investigated the emergence of context-specific plasticity in the auditory system (Diamond and Weinberger, 1989; Cohen et al., 2011), but this work has tended to focus on the adult. Consequently, it is less clear how context-dependent processing emerges during development. In this respect, developmental studies of recurring hearing loss may therefore provide a useful tool for future work.

### Neural traces of context-dependent plasticity

In the specific case of spatial hearing, a key goal for future research will be to characterize the neural circuitry that enables developmental plasticity to be selectively expressed in specific contexts. One possibility is that the brain maintains neural circuits that are appropriate for different sensory contexts but functionally silences circuits that are not appropriate for the prevailing sensory conditions. Consistent with this view, studies in barn owls have shown that prism rearing results in the emergence of novel connections between the ICX and the optic tectum, which provide an anatomical basis for adaptive shifts in ITD tuning that realign the tectal maps of auditory and visual space. These abnormal connections persist following the removal of the prisms and coexist with connections that are appropriate to normal sensory experience, which likely accounts for the capacity of the owls to readapt in later life to the same audiovisual mismatch encountered during development (Linkenhoker and Knudsen, 2002; Linkenhoker et al., 2005).

Interestingly, only one set of connections between the ICX and optic tectum appears to be functionally expressed at any given time, with GABA-mediated inhibition implicated in the selection and stabilization of a particular set of connections (Zheng and Knudsen, 1999, 2001). Similarly, following temporary monocular occlusion during development, mice acquire novel anatomical specializations that persist long after normal vision is restored (Hofer et al., 2009). Although these changes have very little impact on neurophysiological response properties under conditions of normal vision, they nevertheless appear to facilitate more rapid and extensive changes in the visual cortex following a subsequent period of monocular occlusion later in life (Hofer et al., 2006, 2009).

In this way, prior experience can produce changes in neural circuitry that appear to be functionally silenced unless the brain is exposed to specific sensory conditions. Although sensory systems may require relatively long periods of time to switch on functionally silenced circuits, it is equally possible that the brain might learn to rapidly transition between using different circuits. This is likely to be particularly true in situations, such as a recurring hearing loss in one ear, where the auditory system has plenty of experience in switching between different acoustical conditions (Keating et al., 2013a).

However, whilst a recurring hearing loss can produce dramatic changes in auditory input, acoustical conditions can also vary, albeit to a lesser extent, in naturalistic environments. Context-dependent updating of spatial processing is therefore likely to be a general feature of the auditory system, even in individuals without a history of hearing loss. Consistent with this view, two recent studies have shown that the mature auditory system can undergo rapid changes in binaural spatial processing in response to prior acoustical stimulation. In one of these studies, shifts in ILD sensitivity were observed in the ICc as a function of prior stimulus statistics (Dahmen et al., 2010), whilst the other demonstrated GABA_B_ receptor-mediated adaptation in the population code for ITDs found in the MSO (Stange et al., 2013). Although dynamic processing of binaural cues cannot account for the context-dependent plasticity induced by unilateral hearing loss in developing animals (Keating et al., 2013a), similar principles may be involved. Indeed, the adaptive mechanisms that have been observed in normally reared adults may themselves be plastic. In this way, adapting to a recurring hearing loss, either in development or adulthood, may utilize mechanisms that contribute to flexible processing of acoustical inputs under normal hearing conditions. Consequently, studies of recurring hearing loss may provide important insights into normal as well as abnormal adaptive processes in the brain.

## Conclusions

Studies of asymmetric hearing loss have shown that the mechanisms underlying spatial hearing are remarkably plastic during development, with behavioral adaptation in response to altered sensory inputs reported in different species. The underlying basis for adaptation to asymmetric hearing loss appears, however, to vary across species. Thus, barn owls can learn with experience to use altered binaural spatial cues, whereas mammals appear to become more dependent on cues that remain intact and less on those that change in value as a result of the hearing loss. Further research is therefore needed to unravel the reasons for this difference. In addition, whilst the neural basis for behavioral plasticity has been well characterized in barn owls, corresponding studies in mammals are still at a comparatively early stage, highlighting the need for further work in this area.

Although this capacity for change enables the auditory system to adapt to abnormal acoustical inputs, it also represents a major source of developmental vulnerability. In many cases, prolonged periods of hearing loss may lead to amblyaudia, a condition in which the auditory system is unable to fully exploit acoustical input provided to the affected ear if normal hearing is subsequently restored. To the extent that binaural spatial hearing is important for speech comprehension in noisy, naturalistic environments, this may have secondary effects on linguistic, cognitive, social and educational development. It is therefore important to identify individuals who are particularly at risk and identify appropriate strategies for clinical intervention.

In this respect, a key finding to emerge from studies of asymmetric hearing loss is that spatial hearing may be particularly labile early in development. The timing and duration of hearing loss, as well as any necessary clinical intervention, are therefore of considerable importance. Although the stability of hearing loss is likely to play a similarly important role in determining whether intervention is necessary, as well as guiding how intervention might be successfully achieved, this issue has received much less attention in the experimental literature. Recent work, however, suggests that brief intermittent periods of normal hearing may play a protective role, reducing the longer term effects on auditory function when normal inputs become available. In this way, recurring forms of hearing loss may be less damaging than more stable deficits in hearing. Consequently, for individuals who experience a stable hearing loss in one ear, the provision of even relatively brief, intermittent periods of balanced hearing may help to protect against amblyaudia.

Investigation of how the auditory system responds to recurring hearing loss is likely to provide additional insight into a fundamental aspect of neural processing, namely the mechanisms that enable sensory inputs to be processed differently depending on the context in which they occur. In this respect, the context-dependent cue integration revealed by these studies may represent one of the mechanisms through which the auditory system maintains stable and efficient representations of auditory space in different acoustical environments.

To the extent that auditory processing and perception are influenced by sensory context, research in this field may also have important implications for rehabilitation strategies following hearing loss. It has been suggested, for example, that cochlear implants could make use of signal processing strategies that are optimized for specific acoustical contexts, and switch between those strategies depending on the prevailing acoustical conditions (Hu and Loizou, 2010). However, in addition to implementing context-dependence at the level of the device itself, stimulation and rehabilitation strategies might also be designed to exploit the ability of the auditory system to process identical inputs in different ways depending on the context in which they occur. In this way, it may be possible to leverage context-dependent processing for improving perceptual abilities following hearing loss.

### Conflict of interest statement

The authors declare that the research was conducted in the absence of any commercial or financial relationships that could be construed as a potential conflict of interest.
